# Defects in the *C. elegans* acyl-CoA Synthase, *acs-3*, and Nuclear Hormone Receptor, *nhr-25*, Cause Sensitivity to Distinct, but Overlapping Stresses

**DOI:** 10.1371/journal.pone.0092552

**Published:** 2014-03-20

**Authors:** Jordan D. Ward, Brendan Mullaney, Benjamin J. Schiller, Le D. He, Sarah E. Petnic, Carole Couillault, Nathalie Pujol, Teresita U. Bernal, Marc R. Van Gilst, Kaveh Ashrafi, Jonathan J. Ewbank, Keith R. Yamamoto

**Affiliations:** 1 Department of Cellular and Molecular Pharmacology, University of California San Francisco, San Francisco, California, United States of America; 2 Department of Physiology, University of California San Francisco, San Francisco, California, United States of America; 3 Centre d'Immunologie de Marseille-Luminy (CIML), UM2 Aix-Marseille Université, Marseille, France; 4 Institut National de la Santé et de la Recherche Médicale (INSERM), Marseille, France; 5 Centre National de la Recherche Scientifique (CNRS), UMR7280, Marseille, France; 6 Division of Basic Sciences, Fred Hutchinson Cancer Research Center, Seattle, Washington, United States of America; CSIR-Central Drug Research Institute, India

## Abstract

Metazoan transcription factors control distinct networks of genes in specific tissues, yet understanding how these networks are integrated into physiology, development, and homeostasis remains challenging. Inactivation of the nuclear hormone receptor *nhr-25* ameliorates developmental and metabolic phenotypes associated with loss of function of an acyl-CoA synthetase gene, *acs-3*. ACS-3 activity prevents aberrantly high NHR-25 activity. Here, we investigated this relationship further by examining gene expression patterns following *acs-3* and *nhr-25* inactivation. Unexpectedly, we found that the *acs-3* mutation or *nhr-25* RNAi resulted in similar transcriptomes with enrichment in innate immunity and stress response gene expression. Mutants of either gene exhibited distinct sensitivities to pathogens and environmental stresses. Only *nhr-25* was required for wild-type levels of resistance to the bacterial pathogen *P. aeruginosa* and only *acs-3* was required for wild-type levels of resistance to osmotic stress and the oxidative stress generator, juglone. Inactivation of either *acs-3* or *nhr-25* compromised lifespan and resistance to the fungal pathogen *D. coniospora*. Double mutants exhibited more severe defects in the lifespan and *P. aeruginosa* assays, but were similar to the single mutants in other assays. Finally, *acs-3* mutants displayed defects in their epidermal surface barrier, potentially accounting for the observed sensitivities. Together, these data indicate that inactivation of either *acs-3* or *nhr-25* causes stress sensitivity and increased expression of innate immunity/stress genes, most likely by different mechanisms. Elevated expression of these immune/stress genes appears to abrogate the transcriptional signatures relevant to metabolism and development.

## Introduction

Metazoan transcription factors regulate distinct networks of genes in specific cells and tissues to control developmental and homeostatic programs. Understanding how these gene regulatory networks regulate organism development, physiology and homeostasis is a challenging problem. The powerful genetics, compact genome, and simple tissues of *C. elegans* make it an excellent model system in which to study combinatorial regulation of transcription.

The nuclear hormone receptor, NHR-25, is the only *C. elegans* member of the conserved NR5 family, which includes mammalian SF-1 and LRH-1 and *Drosophila* Ftz-F1 [1]. NHR-25 is expressed throughout embryogenesis and development and plays roles in embryonic ventral closure, molting, cuticle formation, epithelial asymmetric cell divisions, vulval development, heterochrony, and fat metabolism [1]–[9]. We know several factors with which *nhr-25* genetically interacts: i) the β-catenins *wrm-1* and *sys-1* in gonadal development [2]; ii) the Hox genes, *lin-39* and *nob-1* in vulval development and embryogenesis [3]; iii) the single SUMO gene, *smo-1*, in vulval development [10]; iv) the Period homolog *lin-42* to regulate molting [11]; and v) the heterochronic genes *hbl-1, let-7*, and *lin-29* [8], [12]. Despite considerable knowledge of co-regulators of *nhr-25*, few regulated genes and no direct targets have been identified.

*nhr-25* interacts genetically with *acs-3*, a long chain acyl-CoA synthetase. Based on genetic data and the ability of recombinant NHR-25 to bind phosphoinositides *in vitro* [4], Mullaney *et al.* (2010) proposed a model in which ACS-3 generates a ligand that acts on NHR-25 to restrict its activity [4]. Phosphoinositide synthesis requires fatty acyl-CoAs, which are produced by acyl CoA synthetases, such as ACS-3 [13]. Moreover, the mammalian orthologs of NHR-25 bind similar phosphoinositide species [14], although the physiological significance of that binding is unknown. Loss of *acs-3* activity in *C. elegans* causes increased intestinal lipid uptake, *de novo* fat synthesis, and accumulation of large, neutral lipid-rich intestinal depots [4]. Although expressed in a number of tissues, previous studies indicate that the key site of action for *acs-3* is in the seam cells [4], cells that contributes both to cuticle generation and morphology [15]. Seam cells are the site of action for a number of nuclear hormone receptors including *nhr-25* [4], [16]–[19]. All previously characterized *acs-3* mutant phenotypes are suppressed by inactivation of *nhr-25* [4].

Our initial goal in this study was to determine how *acs-3* impacts *nhr-25*-dependent gene regulation. Through a combination of genetic and gene expression experiments, we uncovered novel, likely distinct, roles for *acs-3* and *nhr-25* in stress resistance and regulation of stress response/innate immune gene expression.

## Results

### *acs-3* mutation and *nhr-25* RNAi produce similar transcriptomes

The suppression of *acs-3* mutant phenotypes by *nhr-25* reduction-of-function supported a model in which loss of *acs-3* causes increased *nhr-25* activity. This model predicts that there should be a class of genes with increased activity following *acs-3* mutation and decreased activity following knockdown of *nhr-25* [4]. To identify this gene class, we performed two-color microarrays on synchronized mid-L4 larvae. We compared wild-type (WT) N2 animals to *acs-3(ft5)*, a loss-of-function allele that causes mislocalization of the ACS-3 protein [4], and L4440 control RNAi to *nhr-25* RNAi. We chose to use *nhr-25(RNAi)* over the *nhr-25 ku217* allele, a hypomorphic point mutation in the DNA-binding domain [3], because the RNAi was phenotypically stronger for molting defects, sterility and embryonic lethality. To identify genes differentially regulated in *acs-3(ft5)* and *nhr-25(RNAi)* animals, we calculated the statistical significance of the difference in expression between wild-type animals and *acs-3(ft5)* mutants, and control RNAi and *nhr-25(RNAi)*. We used stringent cut-offs similar to Pathare *et al.* [20], which generated a high-confidence list at the expense of potentially losing some regulated genes (p-value cutoff 0.001, 5% false discovery rate, fold-change cutoff of two; see Materials and Methods).

**Figure 1:**
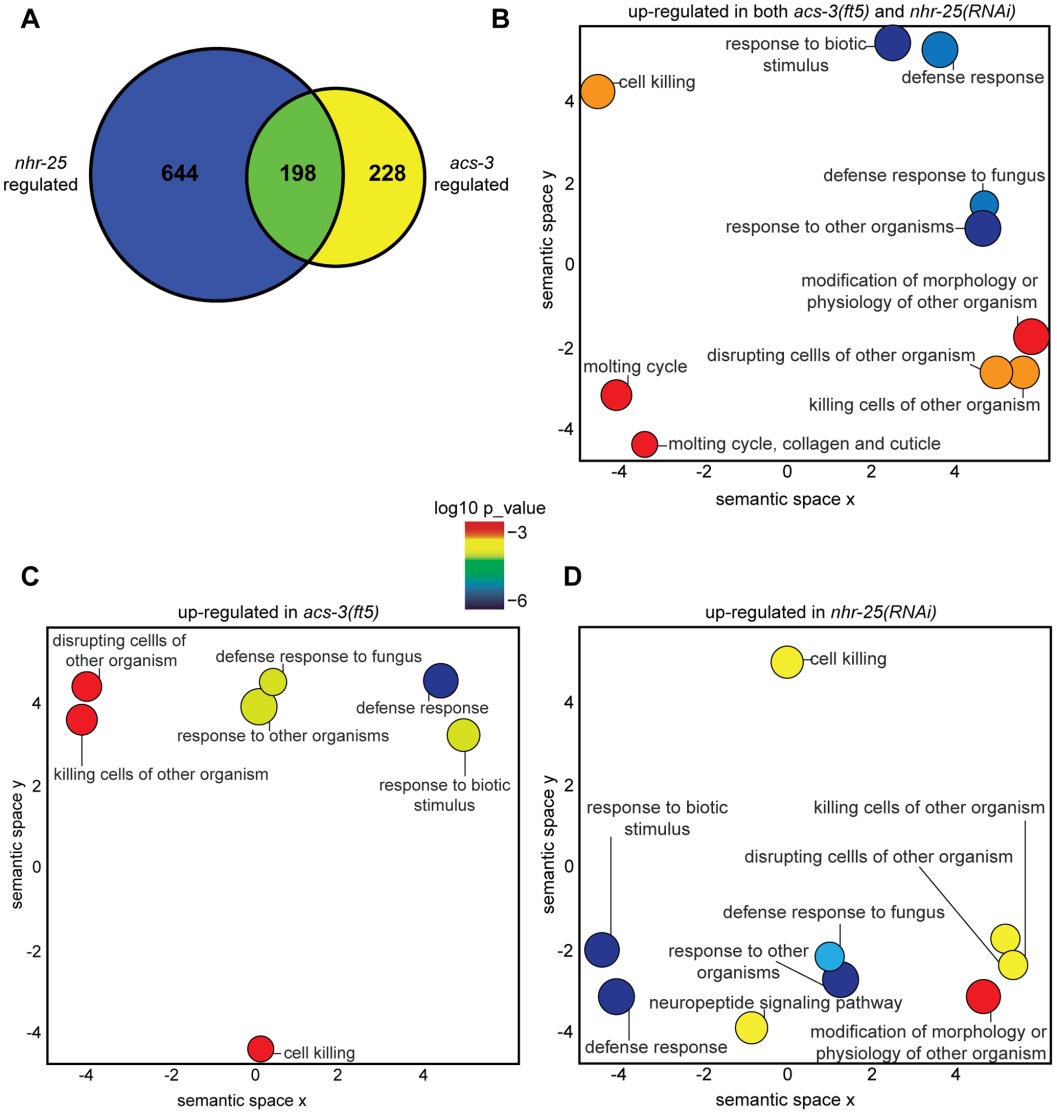
*acs-3* and *nhr-25* regulate similar genes involved in pathogen response and molting. (A) Venn diagram of the overlap between genes differentially up-regulated and down-regulated in the (WT vs *acs-3(ft5)*) and (control vector *(RNAi)* vs *nhr-25(RNAi)*) microarray datasets. Gene lists are provided in Tables S1-4. (B–D) Analysis of enriched biological functions as determined by GORILLA-REVIGO analysis. Enriched biological processes in genes up-regulated in both *acs-3(ft5)* and *nhr-25(RNAi)* animals (B), in *acs-3(ft5)* mutants (C), and in *nhr-25(RNAi)* animals (D) using the GO visualization tool REVIGO [22]. Using this tool, similar functional categories cluster together in two-dimensional space. REVIGO uses a simple clustering algorithm to represent GO data and represents the semantic similarity of the data on the x- and y-axes. Bubble color indicates p-value derived from the Gene Ontology enRIchment anaLysis and visualization tool (GORILLA), and bubble size is proportional to the frequency of GO terms in the Gene Ontology Annotation database. Colors corresponding to log10 p-value are provided in the legend.

We found 426 *acs-3(ft5)* differentially regulated genes, with 275 genes up-regulated in *acs-3(ft5)* mutants (Table S1), and 151 genes down-regulated (Table S2). We found 842 genes differentially regulated in animals treated with *nhr-25(RNAi)* when compared to control RNAi, with 525 up-regulated genes (Table S3), and 317 down-regulated genes (Table S4). To our initial surprise, the *acs-3(ft5)* and *nhr-25(RNAi)* transcriptomes were highly similar (hypergeometric distribution, p<0.0001). Of 426 genes that are differentially expressed between WT animals and *acs-3(ft5)* mutants, and 842 genes that are differentially expressed between control*(RNAi)* and *nhr-25(RNAi)* nematodes, 198 genes were similarly regulated (Figure 1A, 138/275 up- and 60/151 down-regulated genes, or 50.2% and 39.7%, respectively, p<10e-170, Fisher exact test with Bonferroni correction for both).

### *acs-3* and *nhr-25* regulate similar sets of stress response genes

We were interested by the overlap in *acs-3(ft5)* and *nhr-25(RNAi)* gene expression and wanted to examine whether these genes were enriched for particular biological functions by performing gene ontology analysis using the GORILLA and REVIGO tools [21], [22]. In the 151 down-regulated genes of *acs-3* mutants, only *cuticle* and *structural molecule* GO terms were enriched (data not shown), whereas in the 275 up-regulated genes GO terms *defense response, response to biotic stimulus*, and *cell killing* were significantly enriched (Figure 1C; Tables S1 and S2). Performing a similar analysis on the 525 *nhr-25(RNAi)* up-regulated genes, we observed a similar enrichment in the terms *defense response, response to biotic stimulus, cell killing*, as well as *neuropeptide signaling* and *modification of physiology or morphology of other organisms* (Figure 1D; Table S3). Many of the genes in these GO classes overlapped and included *nlp* and *cnc* genes that are up-regulated by pathogen exposure and wounding. The 317 genes down-regulated following *nhr-25(RNAi)* were enriched for *oxidoreductase activity*, *transporter activity, nutrient reservoir*, and *structural component/activity* terms (Figure S1, Table S4). Furthermore, when we performed GO analysis on the 138 genes up-regulated in both *acs-3(ft5)* mutants and *nhr-25(RNAi)* animals, we observed a similar enrichment in *defense response, response to biotic stimulus*, and *cell killing* genes, as well as an enrichment in *molting/cuticle* genes (Figure 1B). The genes populating these GO classes are highlighted in Tables S1–S4.

To further investigate these biological themes, we performed Expression Analysis Systematic Explorer (EASE) analysis to compare our datasets to those in a database manually curated from the literature [23], [24]. Interestingly, the genes up- and down-regulated in *nhr-25(RNAi)* animals most closely resembled genes up- and down-regulated in *acs-3(ft5)* mutants (Tables S5 and S6). Additionally, we observed a significant overlap between genes up-regulated following *nhr-25(RNAi)* and genes up-regulated following *C. elegans* infection with the pathogens *Staphylococcus aureus*, *Photorhabdus luminescens*, or *Drechmeria coniospora*, as well as with genes up-regulated in *dpy-9* and *dpy-10* mutants, which are sensitive to osmotic stress (Table S5). Likewise, we observed a significant overlap between the genes down-regulated following *nhr-25(RNAi)* and genes down-regulated by these same pathogens (Table S6). We observed a similar pattern with genes differentially regulated by: i) the organophosphorus pesticide diazinon; ii) the anthelminthic agent ivermectin; iii) exposure to oxidative, osmotic, heavy metal, and endoplasmic reticulum (ER) stresses; and iv) *osm-8* mutation, which confers sensitivity to osmotic stress (Tables S5 and S6). EASE analysis of the *acs-3(ft5)* mutant up- and down-regulated genes produced results similar to the *nhr-25(RNAi)* EASE data (data not shown). Together, these data suggest that either *acs-3* loss-of-function *(lf)* or *nhr-25* reduction-of-function *(rf)* produced transcriptional responses enriched in the expression of stress response/immunity and molting genes.

### *acs-3* and *nhr-25* mutants express antimicrobial peptide genes in the absence of infection

**Figure 2:**
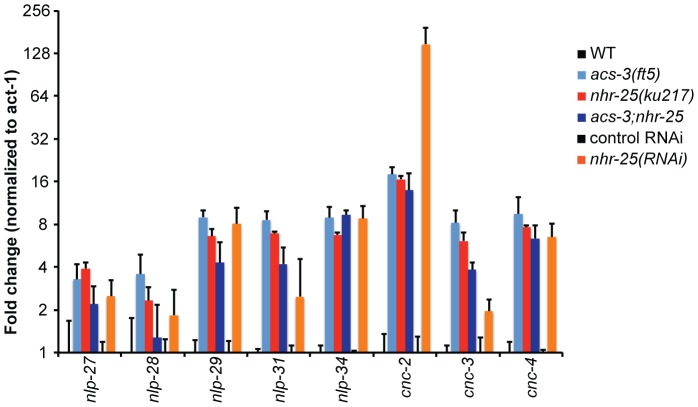
Both *acs-3(lf)* and *nhr-25(rf)* cause activation of antimicrobial peptide genes. qRT-PCR analysis of *nlp* and *cnc* innate immunity genes in L4 stage *acs-3(ft5), nhr-25(ku217)* and *acs-3(ft5);nhr-25(ku217)* mutants relative to WT animals of the same stage and *nhr-25(RNAi)* relative to control control RNAi animals of the same stage. The y-axes provide the fold change and error bars indicate SEM derived from three independent experiments for the WT vs mutant comparison and five independent experiments for control RNAi vs *nhr-25(RNAi)* comparison.

To begin investigating the functional consequences of *acs-3(lf)* and *nhr-25(rf)* on expression of stress and pathogen response genes, we first validated our microarray results by performing qRT-PCR to analyze the expression of a number of candidate defense genes. This analysis was performed using total RNA extracted from synchronized mid-L4 larvae. In agreement with the microarray data, we observed significantly higher expression of the antimicrobial peptide genes *nlp-27, nlp-28, nlp-29, nlp-31, nlp-34, cnc-2, cnc-3*, and *cnc-4* in *acs-3(ft5)* mutants, in the viable *nhr-25(ku217)* hypomorph, and following *nhr-25(RNAi)* (Figure 2). Reduction of *nhr-25* function in the mutant or by RNAi resulted in similar induction of many genes, but we observed higher expression of *cnc-2* in the *nhr-25(RNAi)* animals and higher expression of *nlp-31* and *cnc-3* in *nhr-25(ku217)* animals. These differences may reflect the consequences of reducing *nhr-25* transcript levels by RNAi versus defective NHR-25 DNA binding in the *ku217* mutant. In support of this notion, NHR-25::GFP ChIP-seq data from L2 larvae generated by the model organism ENCyclopedia Of DNA Elements (modENCODE) project [25], [26] found NHR-25 enrichment near the *nlp-31* and *cnc-3* genes (http://modencode.oicr.on.ca/fgb2/gbrowse/worm/). Gene expression data from *nhr-25(rf)* animals matched with NHR-25 ChIP-seq from the same larval stage will be required to further explore whether NHR-25 directly regulates innate immunity genes.

We also tested the effect of simultaneously inactivating *acs-3(ft5)* and *nhr-25(ku217)* by examining pathogen responsive gene expression in an *acs-3(ft5);nhr-25(ku217)* double mutant. We observed similar expression of most assayed antimicrobial peptide genes in the double mutant compared to the two single mutants (Figure 2). However, for *nlp-28, nlp-31*, and *cnc-3*, we observed a moderate reduction in gene expression in the double mutant compared to the single mutants (Figure 2). Thus, loss of *acs-3* activity combined with reduction of *nhr-25* activity did not cause additive increases in innate immunity gene expression for the genes that we tested.

We next used a *nlp-29p::gfp* reporter to validate further the up-regulation of this particular immune target [27] and explore whether there was any stage-specificity to stress response/innate immune gene expression in *acs-3(lf)* or *nhr-25(rf)* animals. This *nlp-29p*::*gfp* reporter has been shown to respond to respond to a range of stresses, such as bacterial and fungal expression, wounding, and osmotic stress [27]–[29]. When this reporter was introduced into *acs-3(ft5)* and *nhr-25(ku217)* mutants, we observed a marked increase in GFP fluorescence (Figure S2). Similarly, *nhr-25(RNAi)* also caused strong expression of the *nlp-29p::gfp* reporter (Figure S2). The microarray, qPCR, and *nlp-29p::gfp* reporter data indicated that *acs-3(lf)* and *nhr-25(rf)* lead to expression of immune genes during growth on a standard lab diet of *E. coli* OP50, with strong expression already apparent in L4 stage larvae. To determine when the *nlp-29p::gfp* reporter is expressed during development we used a COPAS biosorter, which can separate animals on the basis of both size and fluorescence. Using this approach, we examined *acs-3(ft5)* and *nhr-25(ku217)* mutants for elevated *nlp-29p::gfp* reporter expression during development. We grew mixed stage populations of animals and scored both animal size (measured by time of flight (TOF)) and intensity of the *nlp-29p::gfp* reporter. Developmental stage was inferred by TOF: L1 = <200; L2–L3 = 200–400; L4s = 400–600; adults = 600–800. *acs-3(ft5)* mutants exhibited reporter activity early in development (L1 animals), and maintained a sustained 3.5–5.5-fold increase in activity relative to WT animals over the course of their life (Figure S2). *nhr-25(RNAi)*, and to a lesser extent *nhr-25(ku217)*, caused elevated reporter activity early in development (L1 animals), and expression of the reporter was observed throughout development with reporter activity declining in adults (Figure S2). Together, the microarray, qPCR and *nlp-29p::gfp* reporter data indicate that either *acs-3(lf)* or *nhr-25*(*rf*) produced aberrant activation of stress response/innate immune genes.

### *acs-3(ft5)* and *nhr-25(ku217)* mutants exhibit differential stress sensitivities

**Figure 3:**
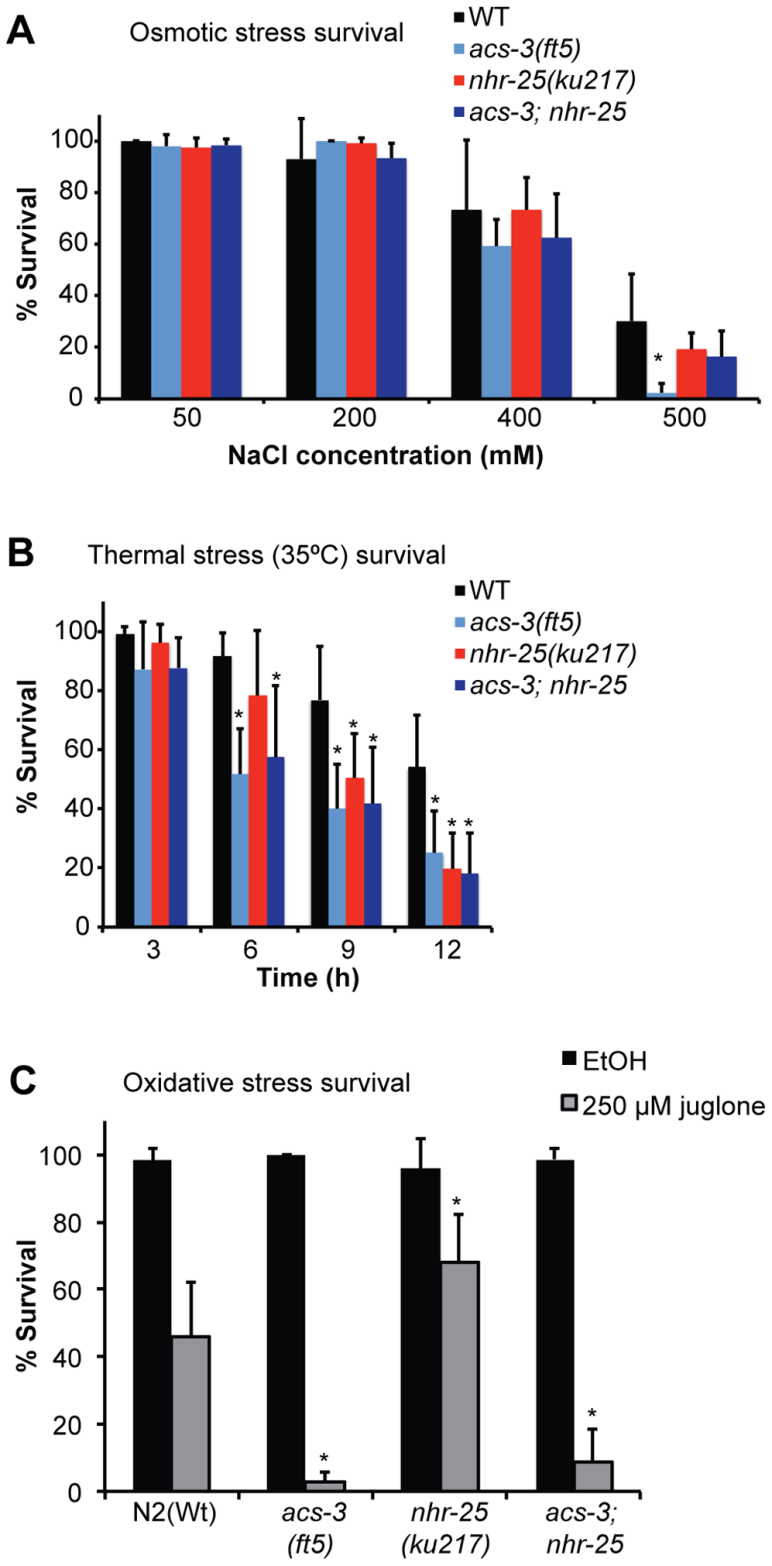
*acs-3(ft5)* and *nhr-25(ku217)* mutants are sensitive to distinct, but overlapping stresses. Sensitivity of L4 larvae of the indicated strains to osmotic stress (A), 35°C thermal stress (B), and oxidative stress (C). For osmotic stress assays (A), animals were scored for viability 24 hours after being placed on plates containing the indicated concentration of salt (50–500 mM NaCl). For thermal stress assays (B), animals were scored for viability every three hours from 0–12 hours. For oxidative stress assays, animals were either placed in 250 μM juglone or an equal amount of ethanol vehicle control for 20 minutes. The animals were then washed onto OP50 plates, allowed to recover for 18 hours, and then scored for viability. Asterisks indicate a statistically significant difference in survival relative to WT controls (two tailed T-test, p<0.05). Detailed statistical analyses, including all pair-wise comparisons are presented in Figure S7.

Given the gene expression signatures of *acs-3(lf)* and *nhr-25(rf)* animals, we sought to distinguish the possibilities that these animals may have either defects that make them sensitive to a range of stresses, or that the stress response/innate immune gene expressions may confer stress resistance, relative to WT animals. Indeed, in a screen for mutants that constitutively upregulate the *nlp-29p::gfp* reporter, Lee *et al.* (2010) identified several mutants that were resistant to osmotic stress [29]. We first examined the roles of *acs-3* and *nhr-25* in response to osmotic stress. Because *nhr-25(RNAi)* causes strong molting defects, potentially confounding functional analyses, we used the *nhr-25(ku217)* hypomorph. L4 larvae were placed on seeded NGM plates assaying an osmolarity range from 50–500 mM NaCl and viability was scored 24 hours later. *acs-3(ft5)* mutants exhibited WT responses to moderate osmotic stress (200–400 mM NaCl), and a mild but significant sensitivity to high osmotic stress (Figure 3A, Table S7A; 500 mM NaCl). *nhr-25(ku217)* mutants were not significantly more sensitive to osmotic stress than WT controls (Figure 3A, Table S7A). Moreover, consistent with developmental and metabolic phenotypes, reduction of function of *nhr-25* alleviated the sensitivity of *acs-3* mutants to high osmotic stress (Figure 3A, Table S7A).

We next examined survival under heat stress (35°C). *acs-3(ft5)* animals were significantly sensitive to heat stress from the six hour timepoint onwards (Figure 3B, Table S7B) while *nhr-25(ku217)* mutants exhibited sensitivity at the 9 and 12 hour timepoints (Figure 3B, Table S7B). *acs-3(ft5)*; *nhr-25(ku217)* double mutants had a similar sensitivity as *acs-3(ft)* mutants (Figure 3B, Table S7B). Thus, unlike the osmotic stress, heat sensitivity of *acs-3* mutants was not dependent on *nhr-25*.

To examine the responses to oxidative stress, we treated WT and mutant animals with the superoxide anion generator, juglone [30], [31]. Following acute exposure to 250 μM juglone or an ethanol control, animals were plated on seeded OP50 plates and viability was scored 18 hours later [32]. As in the case of thermal sensitivity, *acs-3(ft5)* and *acs-3(ft5);nhr-25(ku217)* were hypersensitive to juglone compared to WT animals (Figure 3C, Table S7C). *nhr-25(ku217)* mutants, in contrast, were slightly resistant to juglone (Figure 3C, Table S7C).

**Figure 4:**
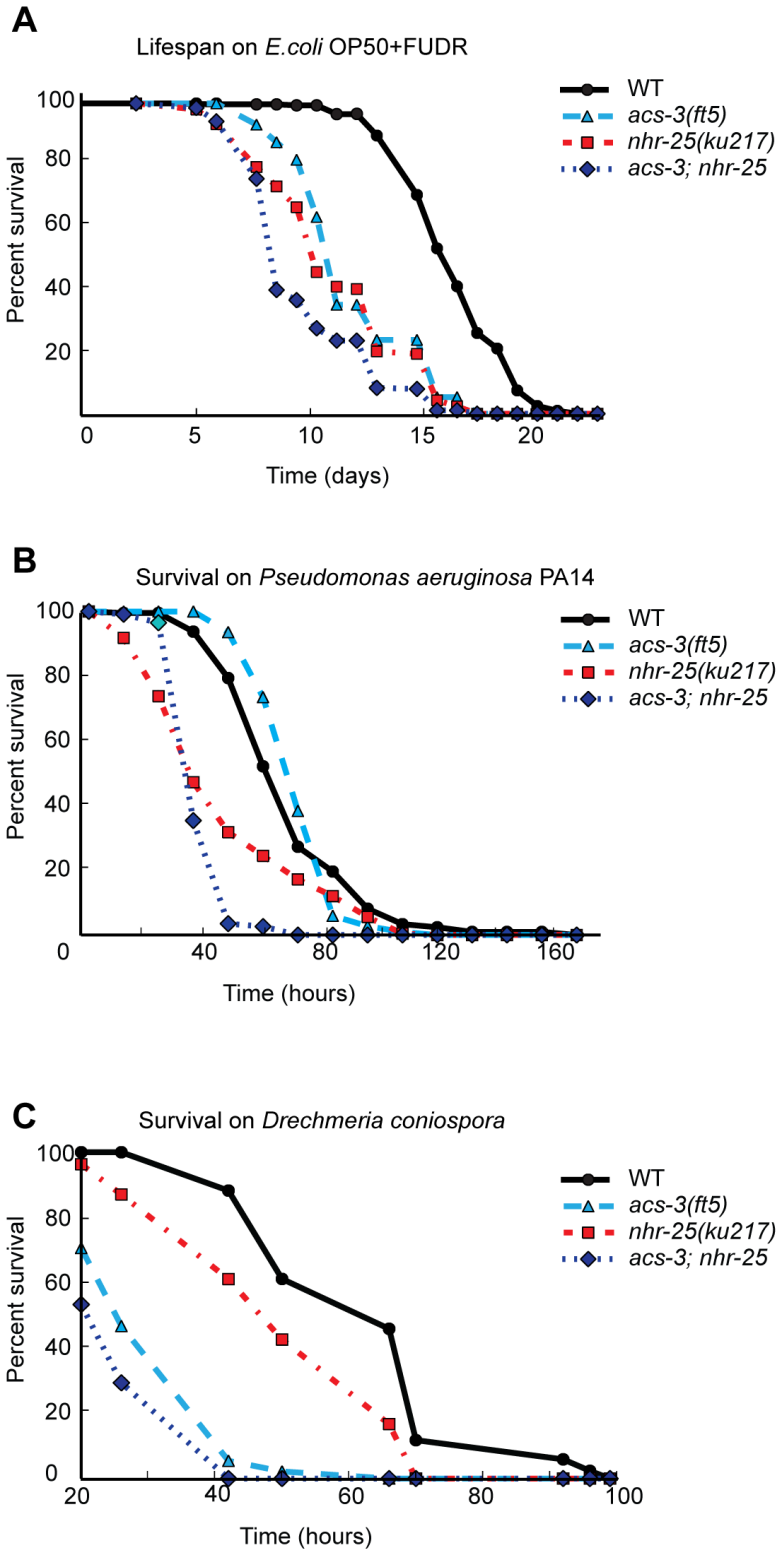
*acs-3(ft5)* and *nhr-25(ku217)* mutants have reduced lifespans and are hypersensitive to *D. coniospora* and *P. aerugninosa*, respectively. (A) Lifespan of the indicated strains on plates supplemented with FUDR and seeded OP50. All lifespan curves are significantly different from one another [n>100; p<0.0001, log-rank (Mantel-Cox) test]. B) Survival of L4 larvae of the indicated genotype on *Pseudomonas aeruginosa* strain PA14. All lifespan curves are significantly different from one another [n>100; p<4.40E-05, log-rank (Mantel-Cox)], except WT and *acs-3(ft5)* [n>100; p = 0.33, log-rank (Mantel-Cox) test]. (C) Survival of L4 larvae of the indicated genotype on *D. coniospora*. The graph is representative of two experiments showing the same results. All strain lifespans are significantly different from one another ([n>100; p≤0.0202, log-rank (Mantel-Cox) test], except *acs-3(ft5)* and *acs-3(ft5);nhr-25 (ku217)* [n>100; p = 0.38, log-rank (Mantel-Cox) test]. Detailed statistical analyses are presented in Figure S8.

As *acs-3* and *nhr-25* mutants were both stress sensitive, we examined their lifespans as a general measure of animal fitness. Plates were supplemented with 5-fluoro-2-deoxyuridine (FUDR) to prevent progeny development, as the vulval defects in *nhr-25(ku217)* mutants lead to death that is due to hatching of progeny within the mother [3]. *acs-3* mutants exhibited a significantly shorter lifespan on *E. coli* OP50, as compared to WT animals (Figure 3A Table S8A) *acs-3(ft5)* mutants also had a reduced brood size (WT = 226±32, *acs-3(ft5)* = 182±27; two-tailed T-test p = 0.008). *nhr-25(ku217)* mutants had a reduced lifespan under these conditions as well (Figure 3A Table S8A). *acs-3; nhr-25* double mutants exhibited a significant decrease in lifespan on OP50+FUDR relative to the single mutants (Figure 4A Table S8A).

*E. coli* OP50 has been reported to be weakly pathogenic for *C. elegans* [33]. We therefore tested whether *acs-3* and *nhr-25* mutants were generally pathogen sensitive, or whether they exhibited any specificity. We first tested sensitivity to the Gram-negative bacterial pathogen *Pseudomonas aeruginosa* strain PA14, which kills animals through either toxin production (fast killing) or intestinal infection (slow killing) [34], [35]. *acs-3* mutants exhibited a WT sensitivity to *P. aeruginosa* in a slow killing assay (Figure 4B, Table S8B), whereas *nhr-25(ku217)* mutants were hypersensitive relative to WT animals (Figure 3B, Table S8B). *acs-3;nhr-25* double mutants were even more sensitive than *nhr-25(ku217)* single mutants (Figure 4B, Table S8B).

Since *acs-3* mutants exhibited wild-type sensitivity to *Pseudomonas aeruginosa*, we next examined the role of *acs-3* and *nhr-25* in the response to *Drechmeria coniospora*, a natural pathogen of *C. elegans*. We chose *D. coniospora* because infection with this nematophagous fungus induces the expression of the *nlp* and *cnc* genes that are deregulated in *nhr-25(RNAi)* and *acs-3(ft5)* transcriptomes, and because these antimicrobial peptide genes contribute to host resistance to infection [28], [36]. Both *acs-3(ft5)* and *nhr-25(ku217)* animals were hypersensitive to *D. coniospora* (Figure 4C, Table S8C and D). The hypersensitivity of *acs-3;nhr-25* double mutants to *D. coniospora* was not significantly greater than *acs-3(ft5)* single mutants (Figure 4C, Table S8C and D).

Collectively, the functional studies indicated that *acs-3(lf)* and *nhr-25(rf)* animals were susceptible to various stresses and pathogens. In the context of these stress conditions, there was no obvious nor consistent genetic regulatory relationship between *acs-3* and *nhr-25*. Given that *acs-3* and *nhr-25* mutants were susceptible to different stresses, our findings were most consistent with the notion that susceptibilities of the two mutants had distinct defects.

### *acs-3(ft5)* mutants have a weak cuticle barrier phenotype

**Figure 5:**
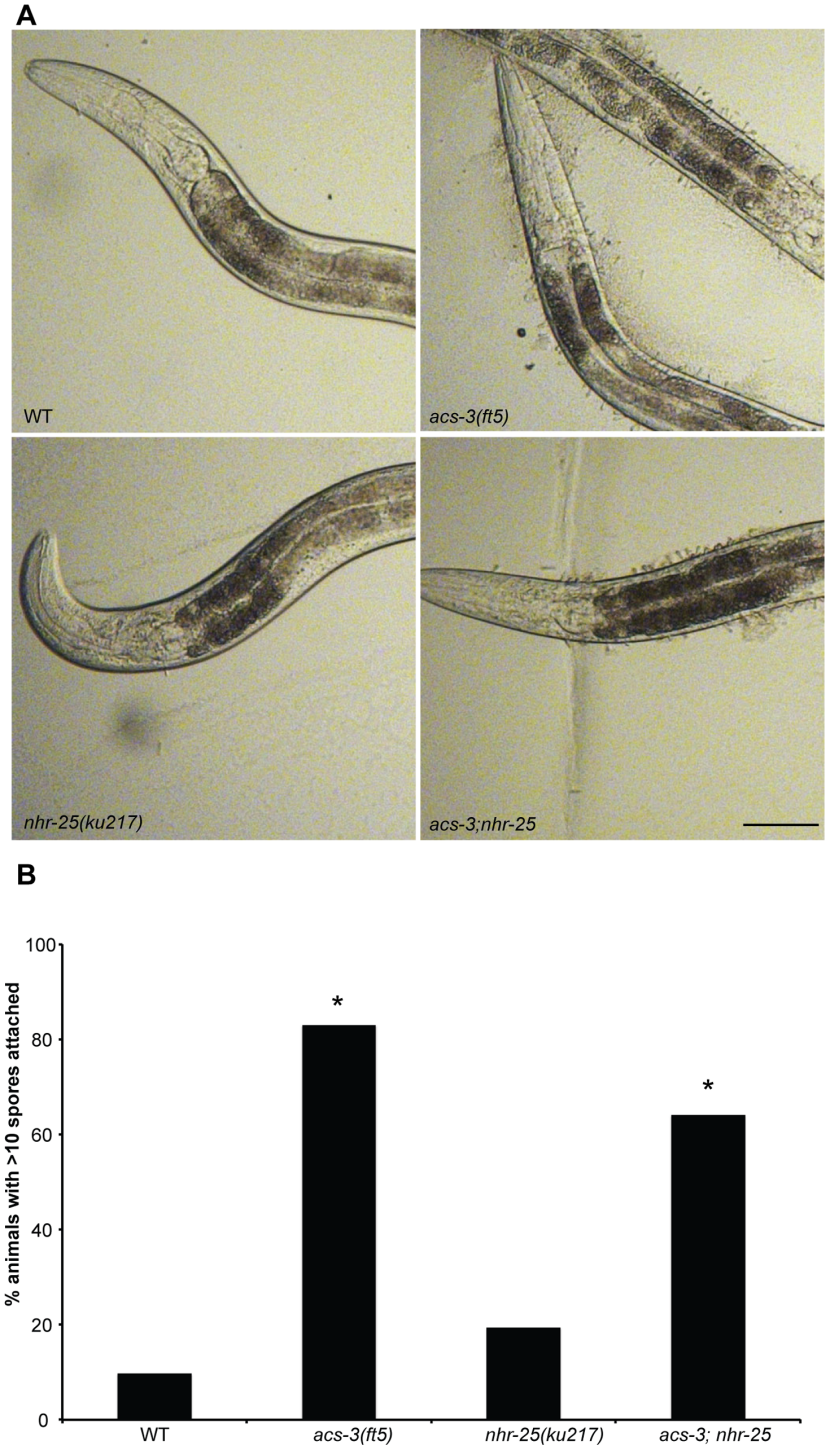
*acs-3(ft5)* mutants have an increase in *D. coniospora* spore adherence. (A) Representative images of *D. coniospora* spore adhesion to L4 larvae of the indicated strains. Images are from an experiment where spores were diluted five-fold. Scale bar = 50 μm (B) Percentage of animals of the indicated genotypes with more than ten spores attached. Asterisks indicate a statistically significant difference in spore attachment relative to wild-type controls (two tailed T-test, p<0.05). Detailed statistical analyses, including all pair-wise comparisons are presented in Table S9. Three independent biological replicates were performed.

We next sought to explore the underlying physiological defects in *acs-3(lf)* and *nhr-25(rf)* animals that could account for the observed stress sensitivities and gene expression profiles. Infection by *D. coniospora* starts with the binding of spores to the nematode cuticle. The cuticle is surrounded by a trilaminar structure known as the epicuticle and evidence from other nematodes suggests that this is a lipid layer [37]–[39]. Surrounding this epicuticle is a surface coat containing carbohydrate and mucin-like proteins [40]–[42]. The increased sensitivity of *acs-*3 or *nhr-25* mutants to *D. coniospora* could be due to alteration of lipid coat composition or structural disruption leading to increased spore attachment. We therefore tested whether *acs-3* or *nhr-25* played roles in preventing *D. coniospora* spore attachment. We did not observe a significant effect using undiluted spore solution (Table S9A). However, upon five-fold dilution of the spore solution we observed a significant increase in spore attachment in both *acs-3(ft5)* and *acs-3;nhr-25* mutants relative to WT animals and *nhr-25(ku217)* mutants (Figure 5, Table S9B). *acs-3(ft5)* mutants and *acs-3;nhr-25* double mutants were not significantly different from one another (Figure 5, Table S9B). These data suggest that the *acs-3(ft5) D. coniospora* sensitivity may be due in part to increased spore attachment and that loss of *acs-3* affected the epithelial barrier.

**Figure 6:**
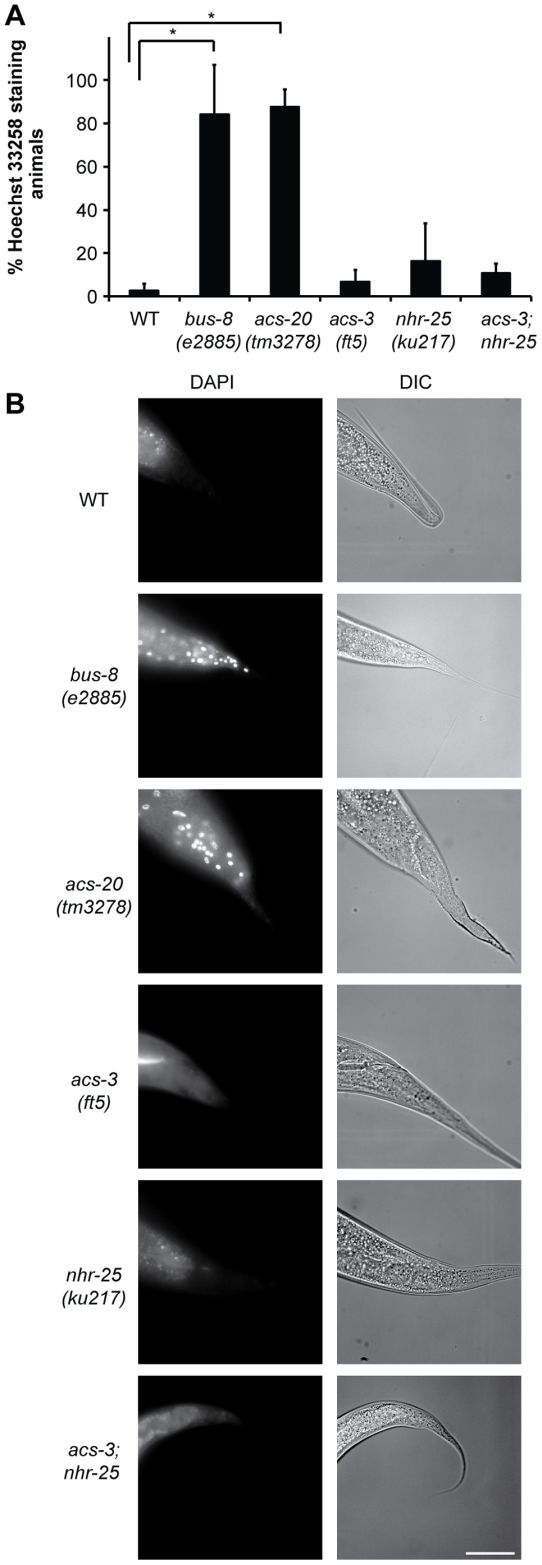
*acs-3(ft5)* and *nhr-25(ku217)* mutant cuticles are not permeable to Hoechst 33258 dye. (A) Permeability to Hoechst 33258 dye. Mixed stage animals of the indicated genotypes were incubated with the membrane impermeable Hoechst 33258 dye and scored for nuclear staining. Error bars represent standard deviation from at least three biological replicates with >50 animals assayed in each experiment (* two-tailed T-test p<0.005). B) Representative DIC and Hoechst staining images for animals of each indicated genotype. Scale bar = 50 μm. Detailed statistical analyses, including all pair-wise comparisons are presented in Figure S10A.

A potential cuticle barrier defect in *acs-3* mutants would be consistent with the emerging role of fat metabolism in the *C. elegans* epidermal barrier. *fasn-1* and *pod-2* catalyze the first two enzymatic steps of *de novo* fatty acid synthesis and RNAi of these genes causes cuticle barrier defects and leads to activation of the *nlp-29p::gfp* reporter [43]. Additionally, loss-of-function of two long chain fatty acid acyl CoA synthases, *acs-20* and *acs-22*, causes defects in cuticle structure and barrier activity [44]. To further test the integrity of the epithelial surface barrier, we incubated mixed-stage animals with the cuticle impermeable, cell membrane permeable Hoechst 33258 dye [45]. In WT animals, the cuticle excludes the dye and prevents staining in non-molting animals. Cuticle permeability was monitored by scoring animals with Hoechst 33258 positive nuclei [45]. Mutations in the glycosyltransferase gene, *bus-8*, cause a compromised epithelial barrier [46] and we observed that 80% of animals stained with Hoechst 33258, essentially identical to that described by Meli *et al.* [47] (Figure 6). Similar to Kage-Nakadai (2010), we observed that *acs-20* mutants also have a compromised epithelial barrier, with approximately 80% of animals exhibiting Hoechst staining (Figure 6). In contrast to *bus-8* and *acs-20* mutants, *acs-3(ft5)* mutants had negligible Hoechst 33258 staining (Figure 6, Table S10A). *nhr-25(ku217)* and *acs-3;nhr-25* mutants had a slight defect in surface barrier function, but the penetrance was weak and only the *acs-3;nhr-25* double mutant was significantly different compared to WT animals (Figure 6, Table S10A). Thus, *acs-3* and *nhr-25* were individually dispensable for exclusion of Hoechst 33258; however, the phenotype of *acs-3;nhr-25* double mutants suggested that they each independently contribute to barrier function.

**Figure 7:**
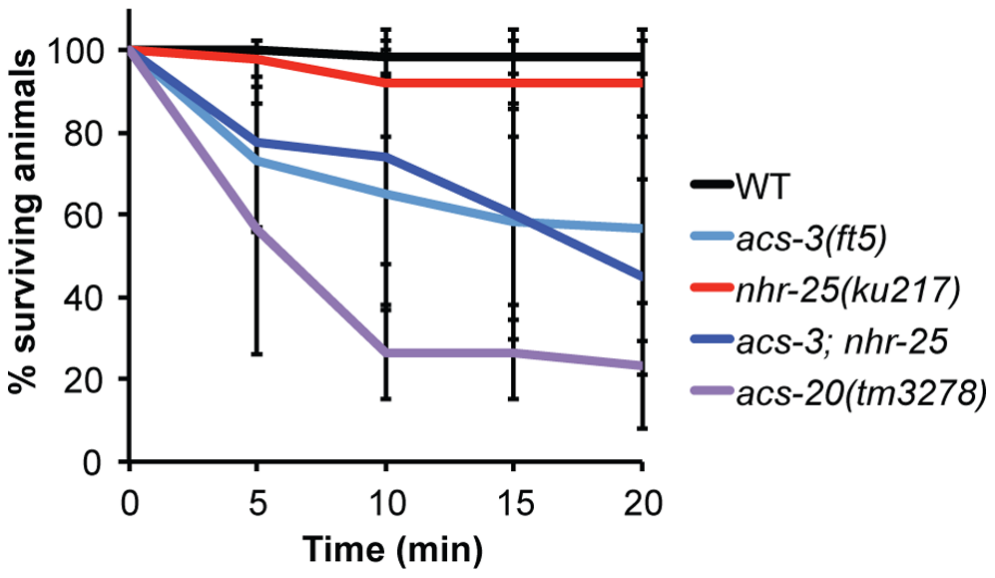
*acs-3(ft5)* mutants are mildly sensitive to hypo-osmotic stress. (A) Animals of the indicated genotype were added to a 50 μl drop of dH_2_0 and viability was assessed every five minutes. Error bars represent standard deviation from 3–5 independent biological replicates with ten animals assayed per experiment (two-tailed T-test for the 20 min timepoint, p<0.05: WT vs *acs-3*, WT vs *acs-20*, WT vs *acs-3;nhr-25*, *nhr-25* vs *acs-3;nhr-25*, and *acs-3 vs acs-20*). Detailed statistical analyses, including all pair-wise comparisons are presented in Figure S10B.

We also tested the surface barrier of *acs-3* and *nhr-25* mutants through hypo-osmotic stress; *acs-20* mutants die rapidly (within 20 minutes) when placed in a drop of water [44]. *acs-3* mutants showed a significant sensitivity to hypo-osmotic stress as approximately 57% of animals reproducibly exploded (Figure 7, Table S10B). Interestingly, the surviving 43% exhibited WT resistance to hypo-osmotic stress throughout the duration of the assay (data not shown). The *acs-3* phenotype was less severe than that of *acs-20* mutants, of which only 23% of mutants survived hypo-osmotic stress (Figure 7). *nhr-25(ku217)* mutants were insensitive to hypo-osmotic stress, and did not affect the *acs-3(ft5)* sensitivity (Figure 7). *acs-20* mutants, and to a lesser extent, *acs-3* mutants also became rigid and paralyzed indicating elevated osmotic stress. WT animals and *nhr-25(ku217)* mutants were not significantly different (Table S10B). Taken together, these results suggest that loss of either *acs-3* or *nhr-25* can cause defects in barrier functions, likely through distinct mechanisms; *acs-3(lf)* and *nhr-25(rf)* animals exhibited different sensitivities in the different assays, and different exacerbations of those sensitivities in the double mutant (Table 1).

**Table 1:** Comparison of the relative phenotypes between strains.

Condition	Severity of phenotype
Stress/immune gene induction	WT *< acs-3 = nhr-25 = acs-3;nhr-25*
Hyper osmotic stress	WT * = nhr-25 = acs-3;nhr-25< acs-3*
Thermal stress	WT *≤ nhr-25< acs-3 = acs-3; nhr-25*
Oxidative stress	*nhr-25>* WT *< acs-3 = acs-3; nhr-25*
Lifespan on OP50	WT *< acs-3< nhr-25< acs-3;nhr-25*
*P. aeruginosa* sensitivity	WT * = acs-3< nhr-25< acs-3;nhr-25*
*D. coniospora* sensitivity	WT *< nhr-25< acs-3 = acs-3;nhr-25*
*D. coniospora* spore adherence	WT * = nhr-25< acs-3 = acs-3;nhr-25*
Hoechst 33258 barrier	WT * = nhr-25 = acs-3 = acs-3;nhr-25*
Hypo-osmotic barrier	WT * = nhr-25< acs-3 = acs-3;nhr-25*

## Discussion

Biochemical and genetic analyses of metabolic phenotypes of *acs-3* mutants indicated that *nhr-25* acts downstream of *acs-3* and that one function of ACS-3 is to inhibit aberrant NHR-25 activity [4]. This conclusion was reached based on analyses of metabolic as well as developmental parameters such as vulval defects and sterility [4]. To better understand the consequences of *acs-3* and *nhr-25* inactivation on metabolism, we originally set out to investigate gene expression in these backgrounds. The previous genetic analyses indicated that there may be genes in *acs-3(ft5)* mutants that are differentially expressed relative to WT animals dependent on *nhr-25* [4]. However, we were surprised to observe a significant overlap in genes differentially expressed in the same direction in both *acs-3(ft5)* mutants and following *nhr-25* RNAi (Figure 1).

While our results might appear at first glance to contradict the regulatory relationship between *acs-3* and *nhr-25* based on the genetic analyses of metabolic and developmental read-outs, we believe in fact that the transcriptional data as they currently stand are inadequate to describe this relationship. First, our microarray results do not rule out the existence of genes transcriptionally up- or down- regulated in *acs-3* mutants dependent on *nhr-25*. Microarrays or RNA-seq data comparing *acs-3* mutant gene expression to *acs-3; nhr-25* double mutants would need to be performed. Furthermore, the metabolic phenotypes of *acs-3* mutants are dependent on expression in the seam cells, which comprise 16–32 cells in L4 larvae [48] As both *acs-3* and *nhr-25* are expressed in cells and tissues other than the seam cells, it may be difficult to detect significant gene expression changes limited to the seam cells. Most significantly, the identities of the up-regulated genes and our functional follow-up studies strongly suggest that both *acs-3* and *nhr-25* mutants suffer from enhanced susceptibility to pathogens and stresses. This pathogen/stress response transcriptional signature might overwhelm any *nhr-25*-dependent gene expression changes caused by *acs-3* inactivation in the seam cells. The findings that *acs-3* and *nhr-25* had distinct sensitivities to various stresses and that some of the defects were exacerbated in the double mutants is most consistent with the notion that different mechanisms underlie the pathogen sensitivity defects of these two mutants. Together, our studies suggest that stress/pathogen sensitivity defects of *acs-3* and *nhr-25* mutations confound the use of transcriptional readouts to search for differentially regulated genes that may account for the metabolic consequences of *acs-3* inactivation.

Interestingly, immune responses are highly integrated with lipid metabolism in both mammals and *Drosophila* [49]. It is unclear whether fatty acids play an active role in innate immunity regulation or signaling in *C. elegans*, although certain gene inactivations that alter metabolism also affect immune responses. For instance, RNAi targeting a number of metabolic genes activated stress reporters and caused aversive behavior on an RNAi bacterial lawn [50]. Inactivation of the fatty acid synthesis genes *fasn-1* and *pod-2* both cause over-expression of antimicrobial peptide genes during growth on *E. coli* OP50; this gene expression depends on WNK-1/GCK-3, a signaling pathway that responds to osmotic stress [29]. Similarly, *acs-3* mutants also upregulate antimicrobial peptide gene expression (Figures 1, 2, S1) and have surface barrier defects (Figures 5 and 7). At present, there is no defined set of metabolic changes that can account for the immune responses and defects caused by these various metabolic enzymes. For instance, while the inactivation of *fasn-1* or *pod-2* critically compromises *de novo* fatty acid synthesis and cause severe fat reduction [43], [51], *acs-3* mutants have both enhanced levels of *de novo* fatty acid synthesis and elevated fat levels [4]. Moreover, while both *fasn-1* and *acs-3* mutants exhibit up-regulation of innate immunity and stress pathways, they exhibit differential responses to pathogen challenge: unlike *fasn-1(fr8)* mutants, which have a slight resistance to *D. coniospora* relative to WT animals [29], *acs-3(ft5)* mutants are hypersensitive (Figure 3C).

Given that alterations in metabolism can have pervasive effects on numerous aspects of animal physiology, it is hardly surprising that metabolic defects may cause up-regulation of stress response pathways. Nevertheless, cuticle defects may be one common feature of mutations that compromise metabolism and pathogen resistance. *acs-20* mutants have aberrations in cuticle structure and a defective surface barrier [44]. Similarly, Lee *et al.* favored the explanation that compromised cuticle or epithelial cell membrane integrity underlie the elevated antimicrobial peptide gene expression in *fasn-1* mutants [29]. In view of the barrier defects that we detected in *acs-3* mutants (Figures 5, 7), this seems the most probable explanation for the pathogen sensitivity and antimicrobial peptide gene expression observed in *acs-3(ft5)* animals. The precise mechanisms that cause *nhr-25(rf)* susceptibility to certain stresses and pathogen responses are unknown. However, defects in the integrity of the cuticle remains a possibility. *nhr-25* is an important transcriptional regulator of the molting genes and *nhr-25(rf)* animals have molting defects. Similar to *nhr-25(rf)*, RNAi inactivation of molting genes cause upregulation of a set of stress and pathogen response genes [50]. *acs-3* mutants do not display obvious molting defects and lack expression of these molting up-regulated stress response markers [50]. These data further support the conclusion that there is likely an inhibitory regulatory relationship between ACS-3 function and NHR-25 activity in certain aspects of metabolism and development, and that certain features associated with inactivation of *acs-3* and *nhr-25* may reflect unrelated processes.

Additional work will be required to characterize the specific defects in *acs-3(lf)* and *nhr-25(rf)* animals that cause stress sensitivity and expression of stress response/innate immune genes. Use of an NHR-25 activity reporter [10] could allow monitoring of NHR-25 output in response to different stresses and genetic backgrounds. Tissue-specific RNA-seq coupled with NHR-25 ChIP-seq would allow determination of the gene regulatory network and determination of direct NHR-25 targets in specific cell-types, such as seam cells. *acs-3* and *nhr-25* function in canonical immune/stress pathways such as the p38/MAP kinase and neuroendocrine TGF-beta signaling cascades [28], [36], [52]–[54] must be assessed. Furthermore, whether *acs-3* or *nhr-25* act with genes that regulate antimicrobial peptide expression specifically in response to fungal infection and epidermal damage (*sta-2, snf-12, nipi-3, dapk-1*) [27], [55], [56], and osmotic stress (*wnk-1-gck-3* signaling) [29] needs to be determined. This work will provide further insight into how NHR-25-regulated genes help ensure proper organism development and physiology and how loss of *acs-3* and *nhr-25* function lead to stress responses and pathogen/stress sensitivity.

## Materials and Methods

### C. elegans strains

*C. elegans* strains were cultured at 25°C for all assays, unless otherwise indicated, to accommodate *nhr-25(ku217)*, which is a temperature sensitive hypomorph [3]. For general strain propagation, animals were grown at 15°C according to standard protocols. The WT strain is the N2 Bristol strain [57]. The following strains were generated for this study: KRY38 *frIs7[nlp-29p::gfp, col-12p::DsRed]* IV; *acs-3(ft5)* V, and KRY39 *frIs7[nlp-29p::gfp, col-12p::DsRed]* IV; *nhr-25(ku217)* X. The following mutant and transgenic strains were used in this study: MH1955 *nhr-25(ku217)* X [3], KQ22 *acs-3(ft5)* V [4], KQ1560 *acs-3(ft5)* V; *nhr-25(ku217)* X [4], IG274 *frIs7[nlp-29p::gfp, col-12p::DsRed]* IV [27], IG348 *fasn-1(fr8)* I; *frIs7[nlp-29p::gfp, col-12p::DsRed]* IV [29]. FX03278 *acs-20(tm3278)* IV [44] was from S. Mitani and the Japanese National Bioresource Project. MT3643 *osm-11(n1604)* X [58] and KU4 *sek-1(km4)* X [52] were obtained from the *Caenorhabditis* Genetics Center (CGC).

### RNA interference

Feeding RNAi was performed as described [59]. RNAi bacteria were grown on LB+Amp+Tet agar. Overnight cultures in LB+Amp were then seeded onto NGM plates with 100 μg/ml ampicillin and 1 mM IPTG and induced overnight at room temperature. The plates were used the following day.

### Biosorter

The quantification of fluorescent reporter gene (GFP or DsRed) expression was performed with the COPAS Biosort (Union Biometrica), as described [27], [36], [53]. Animals were binned by size (TOF) and GFP intensity was averaged.

### Preparation of total nematode mRNA

To obtain synchronized L1 larvae, animals were washed off of three 15 cm plates per genotype. Gravid adults were bleached in alkaline lysis solution, washed five times in M9+gelatin (0.05%), and embryos hatched overnight in the absence of food in M9+gelatin. The following day, animals of the desired genotype were transferred to 15 cm NGM-lite plates seeded with OP50. For RNAi experiments, animals were transferred onto RNAi plates seeded with either L4440 control bacteria or bacteria expressing *nhr-25(RNAi)*. 5,000 animals per plate were used and 15,000-25,000 animals were harvested for each biological replicate. L4 stage animals were harvested, washed three times in M9+gelatin, and flash frozen in liquid nitrogen. For RNA purification, 1 ml of Trizol (Invitrogen) was added to each worm pellet and incubated for 15 minutes at 65°C. RNA purification proceeded according to manufacturer's instructions, except that 100 μl BCP (Molecular Research Center #BP-151) was used as a phase-separation reagent instead of chloroform. RNA cleanup and DNA digestion was performed using the RNeasy kit (Qiagen).

### Microarray analyses

Processing of RNA for microarray analysis, hybridization to Washington University/Genome Sequencing Center *C. elegans* 23 K spotted oligo arrays, and post-hybridization analysis scanning using a GenePix 4000 B scanner (Axon Instruments, Union City, CA) were performed as described [20]. Independent data sets were generated for WT vs *acs-3(ft5)* and control *L4440(RNAi)* vs *nhr-25(RNAi)*. The following “Targets.R” contents were used:

FileNameCy3Cy5

c.elegans_A1vsB1_103.gprvectornhr25

c.elegans_A2vsB2_107.gprvectornhr25

c.elegans_A3vsB3_111.gprvector

c.elegans_C1vsD1_104.gprvectoracs3

c.elegans_C2vsD2_108.gprvectoracs3

c.elegans_C3vsD3_0102.gprvectoracs3

Arrays were normalized with the “maNorm” function from the “marray” package, which uses within-print-tip-group intensity dependent location normalization using the loess function (R “stats” package) and reports log-difference (M) and log-average (A) values.

Pair-wise significance testing (mutant/RNAi vs. wild-type/vector) was performed using the Bioconductor package limma [20] and p-values were initially corrected for multiple testing using the false discovery rate (FDR) method of Benjamini and Hochberg [60]. We defined differential expression as |log2(ratio)| ≥0.848 with the FDR set to 5%, and p-value≤0.001. The microarray data have been deposited in the Gene Expression Omnibus database (http://www.ncbi.nlm.nih.gov/geo/; accession series GSE48605).

Gene names of differentially regulated genes were generated using the WBcel235 release in ENSEMBL BioMart (http://www.ensembl.org/biomart/martview/). Venn Diagrams and the gene overlap between the *acs-3* and *nhr-25* datasets were generated using BioVenn (http://www.cmbi.ru.nl/cdd/biovenn/). Gene Ontology (GO) analysis was performed on the genes regulated by both *acs-3* and *nhr-25* using GOrilla [21]. The GO visualization tool REViGO was used to generate a scatterplot of the functional classification of genes co-regulated by *acs-3* and *nhr-25* [22]. GOrilla is a web interface gene ontology browser that exports data to REVIGO, another web based tool that summarizes ontology data and plots them onto 2D space based on semantic similarity measures [21], [22].

For EASE analysis, the WormBase Converter tool (http://www.ciml.univ-mrs.fr/applications/WB_converter/) was used to convert the gene names from the WBcel235 release to WS230 release gene names [24]. EASE was then performed as previously described [23], [24].

### qRT-PCR analysis

cDNA was synthesized from 2 μg of total RNA in a 40 μl reaction using the iScript cDNA synthesis kit (Bio Rad). Reactions were then diluted to 390 μl with dH_2_0. qRT-PCR reactions were performed as following. 5 μl of the diluted cDNA mix, 1.25 μM of each primer, and 10 μl of 2x Sso Advanced SYBR Green master mix (BioRad) were added to each well. For each experiment, 3–5 biological replicates and 2–3 technical replicates were used. All primer sets were calibrated using serial dilution of cDNA and all qRT-PCR was performed using a CFX Connect machine (BioRad). Each plate contained primers amplifying a reference transcript (*act-1*) and fold-changes of analyzed transcripts are presented relative to this reference gene. *act-1* was chosen as a reference as it showed the smallest variance across the different genotypes and RNAi treatments compared to other commonly used references (*cdc-42, pmp-3*, and *Y45F10D.4* [61], *tba-1* (this study), and *ama-1* [62]) Primer pairs amplifying the *nlp* transcripts are from [28], primers amplifying the *cnc* transcripts are from [36], *act-1* primers are from [62]. Primer sequences are provided in Table S11.

### Stress assays

Hypo-osmotic stress and hyper osmotic stress assays were performed on L4 stage larvae as described [44], [63]. For hyper-osmotic stress assays, plates were brought to room temperature, seeded with OP50, and then sealed with parafilm the day before the experiment. Thermal stress assays were performed similarly to [64]. Briefly, twenty L4 stage larvae were placed on NGM plates seeded with OP50. Plates were placed in a single layer in a 35°C incubator. Viability was assessed every three hours from 0–12 hours. Each strain was assayed in triplicate. Juglone sensitivity experiments were performed on L4 stage larvae as described [32]. For all stress assays, two-tailed T-tests were performed between strains for each timepoint or experimental conditions. All analyses are presented in Table S7.

### Pathogen sensitivity and lifespan assays

Lifespans on OP50 were performed as described [65]. For each genotype, 30 L4 animals were places on each of three 6 cm plates containing 50 μM fluorodeoxyuridine. Assays were carried out at 25°C and animals were transferred to fresh plates every 2–3 days until progeny production ceased. Animals were then transferred to fresh plates only when the OP50 was depleted. Death was scored when animals failed to respond to gentle prodding. *Pseudomonas* PA14 assays were performed as described, with the indicated exceptions [66]. Assays were performed in 6-well plates seeded with PA14. For each genotype, 30 age-matched L4 larvae per well were assayed in triplicate. Viability was scored every 12 hours until all animals were dead. *Drechmeria* survival assays were performed as described [27] on L4 larvae. Lifespan and survival experiments were analyzed using the OASIS online tool which generates Kaplan-Meier survival curves, and statistical data such as p-values from log-rank (Mantel-Cox) [67]. All statistical analyses are presented in Table S8. For *D. coniospora* spore adhesion assays, L4 stage larvae were incubated with either undiluted or 5-fold diluted spore solutions for two hours. Animals were then mounted on a 1% agarose pad and images taken using a Leica MZ16 F (zoom 16∶1) stereomicroscope. Spore attachment was then quantified. Spore concentration is a relative amount not an absolute amount. All strains are exposed to the same spore concentration within an experiment, but the concentration will vary between experiments. Statistical analyses for the spore adhesion experiments are provided in Table S9.

### Cuticle barrier assays

Hoechst 33258 staining was performed similar to Moribe *et al.*, except we used 10 μg/μl of Hoechst 33258 in M9+0.05% gelatin to increase signal. Animals were scored for Hoechst 33258 staining in nuclei. Sensitivity to hypo-osmotic stress was performed as previously described [44]. Statistical analyses for the Hoechst 33258 and hypo-osmotic stress assays are provided in Table S10.

## Supporting Information

Figure S1 **GO terms enriched in** ***nhr-25(RNAi)*** **down-regulated genes.** Enriched biological processes in genes down-regulated in *nhr-25(RNAi)* animals using the GO visualization tool REVIGO [22]. Bubble color indicates p-value derived from the Gene Ontology enRIchment anaLysis and visualization tool (GORILLA), and bubble size is proportional to the frequency of GO terms in the Gene Ontology Annotation database. Colors corresponding to log10 p-value are provided in the legend.
(TIF)

Figure S2 **Quantitative fluorescence analysis of** ***acs-3(ft5), nhr-25(ku217)*** **, and** ***nhr-25(RNAi)*** **.** (A) The Time of Flight (TOF) is a measure of the size of the nematodes and suggestive of the developmental stage. Mixed stage animals of the indicated genotypes and RNAi treatments were passed through a COPAS sorter and TOF and green fluorescence were measured. Animals of different sizes were binned based on TOF and their average GFP signal is indicated on the y-axis. (B) Relative GFP signal of animals of the indicated genotype relative to WT control animals. (C) Relative GFP signal of animals treated with *nhr-25(RNAi)* relative to control RNAi. Approximate developmental stage for each TOF bin is indicated.
(TIF)

Table S1 **Genes up-regulated in** ***acs-3(ft5)*** **mutants.** List of differentially expressed, up-regulated genes in *acs-3(ft5)* mutants compared to WT animals. The data represent the analysis from three independent mRNA isolations and microarray hybridizations. “ID” refers to the identity of individual spots on the arrays, “logFC” represents the log of the fold change, gene public name is from the WS220 release. Genes in the indicated GOrilla gene ontology classes are color coded, as described in the table.
(XLSX)

Table S2 **Genes down-regulated in** ***acs-3(ft5)*** **mutants.** List of differentially expressed, down-regulated genes in *acs-3(ft5)* mutants compared to WT animals. The data represent the analysis from three independent mRNA isolations and microarray hybridizations. “ID” refers to the identity of individual spots on the arrays, “logFC” represents the log of the fold change, gene public name is from the WS220 release. Genes in the indicated GOrilla gene ontology classes are color coded, as described in the table.
(XLSX)

Table S3 **Genes up-regulated following** ***nhr-25(RNAi)*** **.** List of differentially expressed, up-regulated in *nhr-25(RNAi)* animals compared to animals treated with control RNAi. The data represent the analysis from three independent mRNA isolations and microarray hybridizations. “ID” refers to the identity of individual spots on the arrays, “logFC” represents the log of the fold change, gene public name is from the WS220 release. Genes in the indicated GOrilla gene ontology classes are color coded, as described in the table.
(XLSX)

Table S4 **Genes down-regulated following** ***nhr-25(RNAi)*** **.** List of differentially expressed, down-regulated genes in *nhr-25(RNAi)* animals compared to animals treated with control RNAi. The data represent the analysis from three independent mRNA isolations and microarray hybridizations. “ID” refers to the identity of individual spots on the arrays, “logFC” represents the log of the fold change, gene public name is from the WS220 release. Genes in the indicated GOrilla gene ontology classes are color coded, as described in the table.
(XLSX)

Table S5 **EASE analysis of** ***nhr-25(RNAi)*** **up-regulated genes.** EASE analysis of genes up-regulated in *nhr-25(RNAi)* animals compared to control RNAi treated animals. EASE performs statistical analysis to identify significant overrepresentation of functional gene classifications in gene lists. The comparisons to the *acs-3(ft5)* microarray, and to microarray or RNA-seq studies examining responses to pathogens, stress, and pesticides are color coded.
(XLSX)

Table S6 **EASE analysis of** ***nhr-25(RNAi)*** **down-regulated genes.** EASE analysis of genes down-regulated in *nhr-25(RNAi)* animals compared to control RNAi treated animals. EASE performs statistical analysis to identify significant overrepresentation of functional gene classifications in gene lists. The comparisons to the *acs-3(ft5)* microarray, and to microarray or RNA-seq studies examining responses to pathogens, stress, and pesticides are color coded.
(XLSX)

Table S7 **Statistical analyses of stress assays.** Survival data and two tailed T-tests from the osmotic stress (A), thermal stress (B), and oxidative stress (C) experiments.
(DOCX)

Table S8 **Statistical analyses of lifespan and pathogen survival assays.** (A) Statistical analysis of the lifespan of the indicated strains on *E. coli* OP50 containing FUDR. Log rank statistical analyses were performed on three independent experiments. (B) Log rank analyses of survival of the indicated strains on *Pseudomonas aeruginosa* strain PA14; data are from three independent experiments. (C and D) Log rank analyses of survival of the indicated strains on *Drechmeria coniospora*. C and D are analyses from independent biological replicates.
(DOCX)

Table S9 **Statistical analyses of** ***D. coniospora*** **spore adhesion assays.** Spore adhesion data and two tailed T-tests from experiments using undiluted spore solutions (A), and spore solutions diluted five-fold (B). Two independent biological replicates were analyzed in (A), three independent biological replicates were analyzed in (B).
(DOCX)

Table S10 **Statistical analyses of Hoechst 33258 and hypo-osmotic barrier assays.** (A) Hoechst 33258 staining data and two-tailed T-tests from three independent biological replicates. (B) Hypo-osmotic barrier assay data and standard deviation from 3-5 biological replicates. A two-tailed T-test was performed on the data from the 20 minute time point.
(DOCX)

Table S11 **qRT-PCR oligos used in this study.** List of forward and reverse primers used for qRT-PCR experiments. All sequences are displayed in a 5′ to 3′ orientation.
(XLSX)
