# Supplementary figures and images for: Defects in the C. elegans acyl-CoA Synthase, acs-3, and Nuclear Hormone Receptor, nhr-25, Cause Sensitivity to Distinct, but Overlapping Stresses
Source: PLoS One. 2014 Mar 20;9(3):e92552. doi: 10.1371/journal.pone.0092552 (PMC3961378; doi:10.1371/journal.pone.0092552)

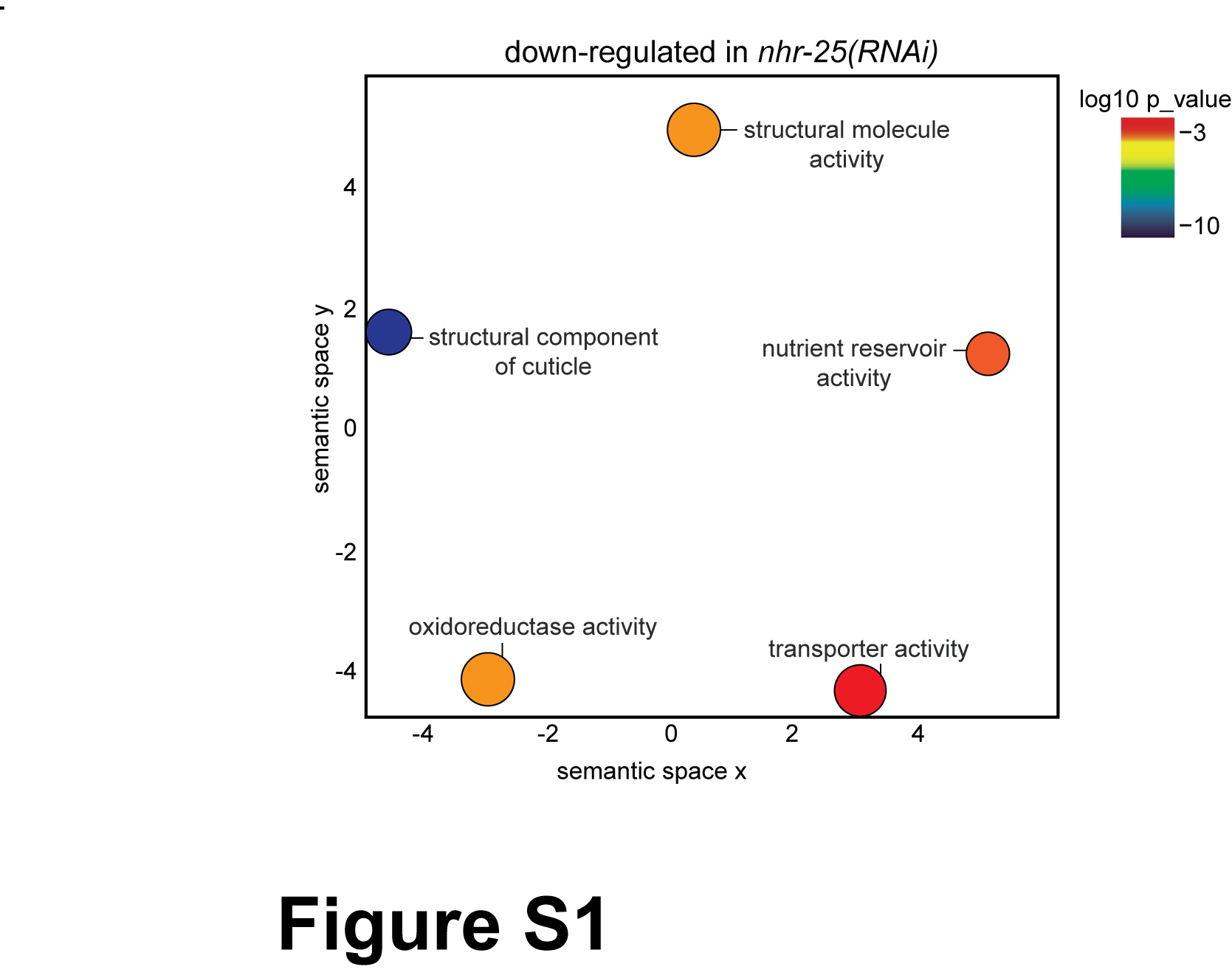

Supplement: Figure S1 — GO terms enriched in nhr-25(RNAi) down-regulated genes. Enriched biological processes in genes down-regulated in nhr-25(RNAi) animals using the GO visualization tool REVIGO [22]. Bubble color indicates p-value derived from the Gene Ontology enRIchment anaLysis and visualization tool (GORILLA), and bubble size is proportional to the frequency of GO terms in the Gene Ontology Annotation database. Colors corresponding to log10 p-value are provided in the legend. (TIF) [file pone.0092552.s001.tif]

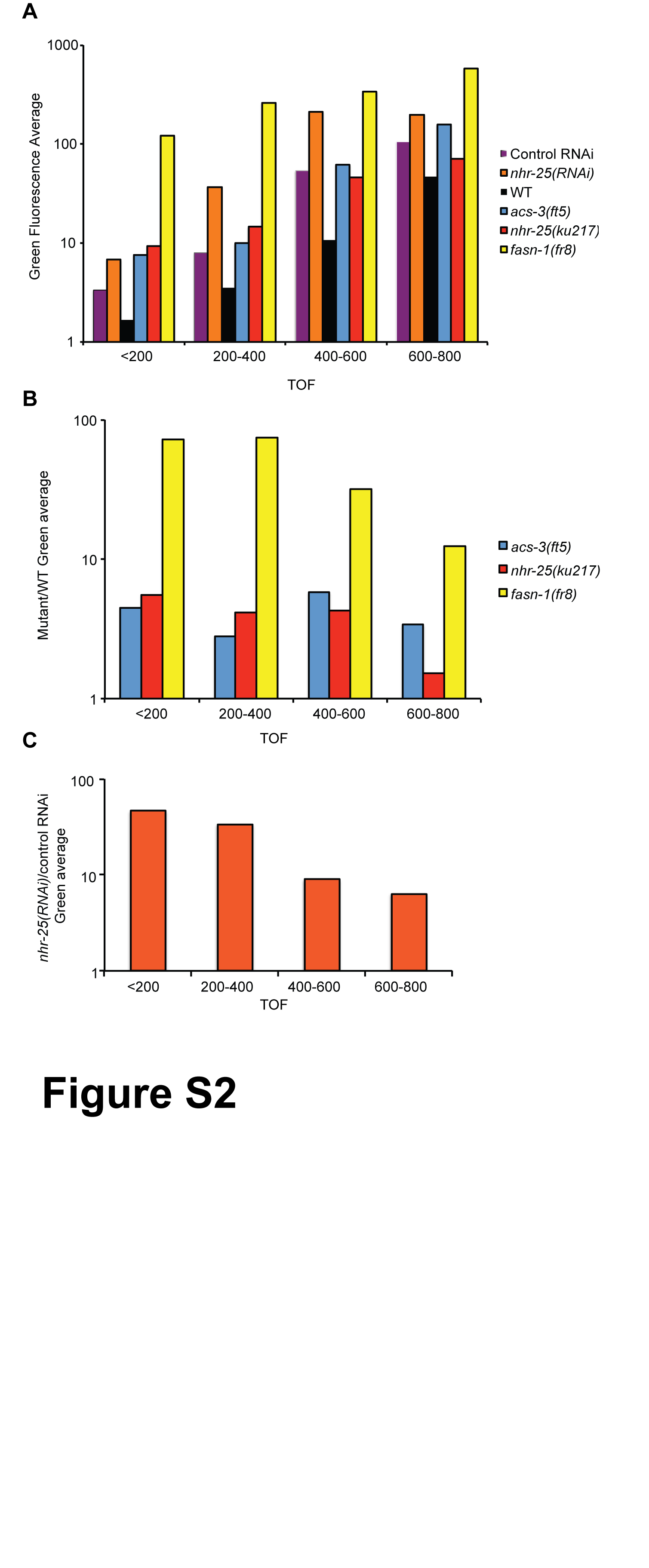

Supplement: Figure S2 — Quantitative fluorescence analysis of acs-3(ft5), nhr-25(ku217) , and nhr-25(RNAi) . (A) The Time of Flight (TOF) is a measure of the size of the nematodes and suggestive of the developmental stage. Mixed stage animals of the indicated genotypes and RNAi treatments were passed through a COPAS sorter and TOF and green fluorescence were measured. Animals of different sizes were binned based on TOF and their average GFP signal is indicated on the y-axis. (B) Relative GFP signal of animals of the indicated genotype relative to WT control animals. (C) Relative GFP signal of animals treated with nhr-25(RNAi) relative to control RNAi. Approximate developmental stage for each TOF bin is indicated. (TIF) [file pone.0092552.s002.tif]
