# Supplementary material for: Defects in the C. elegans acyl-CoA Synthase, acs-3, and Nuclear Hormone Receptor, nhr-25, Cause Sensitivity to Distinct, but Overlapping Stresses
Source: PLoS One. 2014 Mar 20;9(3):e92552. doi: 10.1371/journal.pone.0092552 (PMC3961378; doi:10.1371/journal.pone.0092552)
Supplement: Table S7 — Statistical analyses of stress assays. Survival data and two tailed T-tests from the osmotic stress (A), thermal stress (B), and oxidative stress (C) experiments. (DOCX) [file pone.0092552.s009.docx]

**Table S7. Statistical analyses of stress assays.**

A) Osmotic stress survival data and two-tailed T-tests from three independent experiments

|  | **% Survival** | | | |
| --- | --- | --- | --- | --- |
| **mM NaCl** | **WT** | ***acs-3(ft5)*** | ***nhr-25(ku217)*** | ***acs-3; nhr-25*** |
| 50 | 100.00 | 98.00 | 97.46 | 98.33 |
| 200 | 93.00 | 100.00 | 99.09 | 93.28 |
| 400 | 73.24 | 59.06 | 73.20 | 62.35 |
| 500 | 30.22 | 2.41 | 19.40 | 16.26 |

|  | **Std Dev of % Survival** | | | |
| --- | --- | --- | --- | --- |
| **mM NaCl** | **WT** | ***acs-3(ft5)*** | ***nhr-25(ku217)*** | ***acs-3; nhr-25*** |
| 50 | 0.00 | 4.47 | 3.75 | 2.29 |
| 200 | 15.65 | 0.00 | 2.03 | 5.72 |
| 400 | 26.97 | 10.38 | 12.48 | 17.01 |
| 500 | 18.12 | 3.50 | 6.14 | 10.03 |

| **T-test** | **50 mM** | **200 mM** | **400 mM** | **500 mM** |
| --- | --- | --- | --- | --- |
| WT *vs acs-3* | 3.74E-01 | 3.74E-01 | 4.19E-01 | 2.99E-02 |
| WT *vs nhr-25* | 2.05E-01 | 4.50E-01 | 9.96E-01 | 3.16E-01 |
| WT *vs acs-3;nhr-25* | 1.78E-01 | 9.69E-01 | 5.03E-01 | 2.30E-01 |
|  |  |  |  |  |
| *acs-3 vs nhr-25* | 3.91E-01 | 3.74E-01 | 1.95E-01 | 1.26E-02 |
| *acs-3 vs acs-3;nhr-25* | 1.54E-01 | 5.84E-02 | 6.79E-01 | 1.25E-02 |
|  |  |  |  |  |
| *nhr-25 vs acs-3;nhr-25* | 3.09E-01 | 1.46E-01 | 1.95E-01 | 6.56E-01 |

N/A-no value as results in two conditions are identical (0% survival)

B) Heat-stress (35ºC) survival data and two-tailed T-tests from three independent experiments

|  | **% Survival** | | | |
| --- | --- | --- | --- | --- |
| **Time (h)** | **WT** | ***acs-3(ft5)*** | ***nhr-25(ku217)*** | ***acs-3; nhr-25*** |
| 3 | 99.07 | 87.17 | 96.05 | 87.55 |
| 6 | 91.62 | 51.75 | 78.09 | 57.49 |
| 9 | 76.81 | 39.91 | 50.41 | 41.84 |
| 12 | 54.00 | 24.97 | 19.56 | 18.02 |
|  |  |  |  |  |
|  |  |  |  |  |
|  | **Std Dev of % Survival** | | | |
| **Time (h)** | **WT** | ***acs-3(ft5)*** | ***nhr-25(ku217)*** | ***acs-3; nhr-25*** |
| 3 | 2.27 | 16.21 | 6.51 | 10.28 |
| 6 | 7.98 | 15.28 | 22.10 | 24.11 |
| 9 | 18.01 | 15.11 | 14.96 | 19.11 |
| 12 | 17.60 | 14.36 | 12.14 | 13.88 |

| **T-test** | **3hr** | **6hr** | **9 hr** | **12 hr** |
| --- | --- | --- | --- | --- |
| WT *vs acs-3* | 1.44E-01 | 2.26E-03 | 6.54E-03 | 2.39E-02 |
| WT *vs nhr-25* | 2.03E-01 | 1.16E-01 | 1.46E-02 | 1.75E-02 |
| WT *vs acs-3;nhr-25* | 6.03E-02 | 5.57E-03 | 9.97E-05 | 1.63E-03 |
|  |  |  |  |  |
| *acs-3 vs nhr-25* | 2.33E-01 | 1.66E-02 | 7.19E-02 | 9.36E-01 |
| *acs-3 vs acs-3;nhr-25* | 1.52E-01 | 8.55E-01 | 6.49E-01 | 3.86E-01 |
|  |  |  |  |  |
| *nhr-25 vs acs-3;nhr-25* | 4.70E-02 | 1.88E-02 | 1.44E-01 | 3.73E-01 |

C) Juglone stress survival data and two-tailed T-tests from three independent experiments

|  | **EtOH % Survival** | **Std Dev** | **250 µM juglone % Survival** | **Std Dev** |
| --- | --- | --- | --- | --- |
| WT | 98.46 | 3.44 | 46.11 | 16.15 |
| *acs-3(ft5)* | 100.00 | 0.00 | 2.93 | 2.81 |
| *nhr-25(ku217)* | 96.00 | 8.94 | 67.89 | 14.19 |
| *acs-3(ft5);nhr-25(ku217)* | 98.67 | 2.98 | 8.65 | 9.71 |

|  | T-test P-value |
| --- | --- |
| WT *vs acs-3* | 5.68E-03 |
| WT *vs nhr-25* | 1.20E-02 |
| WT *vs acs-3;nhr-25* | 8.28E-03 |
|  |  |
| *acs-3 vs nhr-25* | 6.90E-04 |
| *acs-3 vs acs-3;nhr-25* | 2.94E-01 |
|  |  |
| *nhr-25 vs acs-3;nhr-25* | 2.15E-04 |
