# Supplementary material for: Defects in the C. elegans acyl-CoA Synthase, acs-3, and Nuclear Hormone Receptor, nhr-25, Cause Sensitivity to Distinct, but Overlapping Stresses
Source: PLoS One. 2014 Mar 20;9(3):e92552. doi: 10.1371/journal.pone.0092552 (PMC3961378; doi:10.1371/journal.pone.0092552)
Supplement: Table S8 — Statistical analyses of lifespan and pathogen survival assays. (A) Statistical analysis of the lifespan of the indicated strains on E. coli OP50 containing FUDR. Log rank statistical analyses were performed on three independent experiments. (B) Log rank analyses of survival of the indicated strains on Pseudomonas aeruginosa strain PA14; data are from three independent experiments. (C and D) Log rank analyses of survival of the indicated strains on Drechmeria coniospora. C and D are analyses from independent biological replicates. (DOCX) [file pone.0092552.s010.docx]

**Table S8. Statistical analyses of lifespan and pathogen survival assays.**

A) *E. coli* OP50+FUDR lifespan. Log-Rank statistical analysis from three independent experiments

|  |  | **Statistics** |  |
| --- | --- | --- | --- |
| **Condition** | **Chi^2** | **P-value** | **Bonferroni P-value** |
| WT *vs acs-3* | 294.24 | p < 0.0001 | p < 0.0001 |
| WT *vs nhr-25* | 319.12 | p < 0.0001 | p < 0.0001 |
| WT *vs acs-3;nhr-25* | 473.36 | p < 0.0001 | p < 0.0001 |
| *acs-3 vs nhr-25* | 6.81 | 0.0091 | 0.0272 |
| *acs-3 vs acs-3;nhr-25* | 76.34 | p < 0.0001 | p < 0.0001 |
| *nhr-25 vs acs-3;nhr-25* | 27.16 | 1.90E-07 | 5.60E-07 |

|  |  | **Restricted mean** | | |
| --- | --- | --- | --- | --- |
| **Strain** | **n** | **Days** | **Std. error** | **95% C.I.** |
| WT | 319 | 17.93 | 0.14 | 17.65 ~ 18.22 |
| *acs-3* | 336 | 12.78 | 0.16 | 12.46 ~ 13.11 |
| *nhr-25* | 254 | 11.97 | 0.22 | 11.53 ~ 12.40 |
| *acs-3;nhr-25* | 276 | 10.41 | 0.18 | 10.05 ~ 10.76 |

B) *Pseudomonas aeruginosa* PA14 survival Log-Rank statistical analysis from three independent experiments

|  |  | **Statistics** |  |
| --- | --- | --- | --- |
| **Condition** | **Chi^2** | **P-value** | **Bonferroni P-value** |
| WT *vs acs-3* | 0.93 | 0.3344 | 1 |
| WT *vs nhr-25* | 50.34 | p < 0.0001 | p < 0.0001 |
| WT *vs acs-3;nhr-25* | 279.6 | p < 0.0001 | p < 0.0001 |
| *acs-3 vs nhr-25* | 62.93 | p < 0.0001 | p < 0.0001 |
| *acs-3 vs acs-3;nhr-25* | 390.87 | p < 0.0001 | p < 0.0001 |
| *nhr-25 vs acs-3;nhr-25* | 16.68 | 4.40E-05 | 0.0001 |

|  |  | **Restricted mean** | | |
| --- | --- | --- | --- | --- |
| **Strain** | **n** | **Hours** | **Std. error** | **95% C.I.** |
| **WT** | 209 | 70.56 | 1.57 | 67.50 ~ 73.63 |
| **acs-3** | 219 | 73.81 | 0.89 | 72.07 ~ 75.55 |
| **nhr-25** | 233 | 48.52 | 1.76 | 45.06 ~ 51.97 |
| **acs-3;nhr-25** | 229 | 40.45 | 0.57 | 39.34 ~ 41.57 |

C) *Drechmeria coniospora* survival Log-Rank statistical analysis biological replicate #1

|  |  | **Statistics** |  |
| --- | --- | --- | --- |
| **Condition** | **Chi^2** | **P-value** | **Bonferroni P-value** |
| WT *vs acs-3* | 57.62 | p < 0.0001 | p < 0.0001 |
| WT *vs nhr-25* | 7.35 | 0.0067 | 0.0202 |
| WT *vs acs-3;nhr-25* | 65.78 | p < 0.0001 | p < 0.0001 |
| *acs-3 vs nhr-25* | 21.87 | 2.90E-06 | 8.80E-06 |
| *acs-3 vs acs-3;nhr-25* | 2.33 | 0.1269 | 0.3808 |
| *nhr-25 vs acs-3;nhr-25* | 25.73 | 3.90E-07 | 1.20E-06 |

|  |  | **Restricted mean** | | |
| --- | --- | --- | --- | --- |
| **Strain** | **n** | **Hours** | **Std. error** | **95% C.I.** |
| WT | 85 | 63.58 | 1.66 | 60.33 ~ 66.83 |
| *acs-3* | 92 | 32.50 | 1.18 | 30.19 ~ 34.81 |
| *nhr-25* | 54 | 52.07 | 2.07 | 48.01 ~ 56.14 |
| *acs-3;nhr-25* | 75 | 27.89 | 1.09 | 25.77 ~ 30.02 |

D) *Drechmeria coniospora* survival Log-Rank statistical analysis biological replicate #2

|  |  | **Statistics** |  |
| --- | --- | --- | --- |
| **Condition** | **Chi^2** | **P-value** | **Bonferroni P-value** |
| WT *vs acs-3* | 75.21 | p < 0.0001 | p < 0.0001 |
| WT *vs nhr-25* | 8.51 | 0.0035 | 0.0106 |
| WT *vs acs-3;nhr-25* | 58.84 | p < 0.0001 | p < 0.0001 |
| *acs-3 vs nhr-25* | 32.88 | 9.80E-09 | 2.90E-08 |
| *acs-3 vs acs-3;nhr-25* | 6.58 | 0.0103 | 0.0309 |
| *nhr-25 vs acs-3;nhr-25* | 20.12 | 7.30E-06 | 2.20E-05 |

|  |  |  |  |  |
| --- | --- | --- | --- | --- |
|  |  | **Restricted mean** | | |
| **Strain** | **n** | **Hours** | **Std. error** | **95% C.I.** |
| WT | 42 | 62.95 | 2.07 | 58.90 ~ 67.01 |
| *acs-3* | 47 | 31.21 | 1.59 | 28.10 ~ 34.32 |
| *nhr-25* | 25 | 52.04 | 2.7 | 46.76 ~ 57.32 |
| *acs-3;nhr-25* | 43 | 33.79 | 1.51 | 30.83 ~ 36.75 |
