# Supplementary material for: Defects in the C. elegans acyl-CoA Synthase, acs-3, and Nuclear Hormone Receptor, nhr-25, Cause Sensitivity to Distinct, but Overlapping Stresses
Source: PLoS One. 2014 Mar 20;9(3):e92552. doi: 10.1371/journal.pone.0092552 (PMC3961378; doi:10.1371/journal.pone.0092552)
Supplement: Table S9 — Statistical analyses of D. coniospora spore adhesion assays. Spore adhesion data and two tailed T-tests from experiments using undiluted spore solutions (A), and spore solutions diluted five-fold (B). Two independent biological replicates were analyzed in (A), three independent biological replicates were analyzed in (B). (DOCX) [file pone.0092552.s011.docx]

**Table S9. Statistical analyses of *D. coniospora* spore adhesion assays.**

A) Undiluted spore adhesion assay data and t-tests from two independent experiments

| **Strain** | **% worms with more than ten spores attached** | **n=** |
| --- | --- | --- |
| WT | 68.08 | 33 |
| *acs-3(ft5)* | 94.44 | 34 |
| *nhr-25(ku217)* | 79.00 | 31 |
| *acs-3; nhr-25* | 100.00 | 39 |

n=number of worms assayed

| **T-test** | **P-value** |
| --- | --- |
| WT vs *acs-3* | 2.57E-01 |
| WT vs *nhr-25* | 2.50E-01 |
| WT vs double | 2.50E-01 |
|  |  |
| *acs-3* vs *nhr-25* | 2.61E-01 |
| *acs-3* vs double | 2.50E-01 |
|  |  |
| *nhr-25* vs double | 1.54E-01 |

B) Five-fold dilution spore adhesion assay t-tests from three independent experiments

| **Strain** | **% worms with more than ten spores attached** | **n=** |
| --- | --- | --- |
| WT | 9.67 | 51 |
| *acs-3(ft5)* | 83.05 | 67 |
| *nhr-25(ku217)* | 19.38 | 57 |
| *acs-3; nhr-25* | 64.09 | 54 |

n=number of worms assayed

| **T-test** | **P-value** |
| --- | --- |
| WT vs *acs-3* | 1.49E-02 |
| WT vs *nhr-25* | 2.17E-01 |
| WT vs double | 5.03E-03 |
|  |  |
| *acs-3* vs *nhr-25* | 3.21E-02 |
| *acs-3* vs double | 7.46E-02 |
|  |  |
| *nhr-25* vs double | 4.02E-02 |
