# Supplementary material for: Defects in the C. elegans acyl-CoA Synthase, acs-3, and Nuclear Hormone Receptor, nhr-25, Cause Sensitivity to Distinct, but Overlapping Stresses
Source: PLoS One. 2014 Mar 20;9(3):e92552. doi: 10.1371/journal.pone.0092552 (PMC3961378; doi:10.1371/journal.pone.0092552)
Supplement: Table S10 — Statistical analyses of Hoechst 33258 and hypo-osmotic barrier assays. (A) Hoechst 33258 staining data and two-tailed T-tests from three independent biological replicates. (B) Hypo-osmotic barrier assay data and standard deviation from 3-5 biological replicates. A two-tailed T-test was performed on the data from the 20 minute time point. (DOCX) [file pone.0092552.s012.docx]

**Table S10. Statistical analyses of Hoechst 33258 and hypo-osmotic barrier assays.**

A) Hoechst staining two-tailed T-tests from three independent experiments

| **Strain** | **% Hoechst positive nuclei** | **Std. Dev** |
| --- | --- | --- |
| WT | 3 | 0.03 |
| *bus-8(e2885)* | 84 | 0.23 |
| *acs-20(tm3278)* | 88 | 0.08 |
| *acs-3(ft5)* | 7 | 0.06 |
| *nhr-25(ku217)* | 16 | 0.18 |
| *acs-3; nhr-25* | 11 | 0.05 |

|  | T-test P-value |
| --- | --- |
| WT vs *bus-8* | 2.44E-03 |
| WT vs *acs-20* | 2.75E-04 |
| WT vs *acs-3* | 8.00E-02 |
| WT vs *nhr-25* | 9.13E-02 |
| WT vs *acs-3 nhr-25* | 4.39E-02 |

B) Hypo-osmotic stress raw data, and standard deviation from 3-5 independent biological replicates. A two-tailed T-test for the 20 minute timepoint is provided.

|  | % Survival over time (min) | | | | |
| --- | --- | --- | --- | --- | --- |
| **Strain** | **0** | **5** | **10** | **15** | **20** |
| WT | 100 | 100.00 | 98.33 | 98.33 | 98.33 |
| *acs-3(ft5)* | 100 | 73.33 | 65.00 | 58.33 | 56.67 |
| *nhr-25(ku217)* | 100 | 98.00 | 92.00 | 92.00 | 92.00 |
| *acs-3; nhr-25* | 100 | 77.50 | 74.00 | 60.00 | 45.00 |
| *acs-20(tm3278)* | 100 | 56.67 | 26.67 | 26.67 | 23.33 |
|  |  |  |  |  |  |
|  | Std Dev of % Survival over time (min) | | | | |
| **Strain** | **0** | **5** | **10** | **15** | **20** |
| WT | - | 0.00 | 4.08 | 4.08 | 4.08 |
| *acs-3(ft5)* | - | 17.51 | 28.11 | 28.58 | 27.33 |
| *nhr-25(ku217)* | - | 4.47 | 13.04 | 13.04 | 13.04 |
| *acs-3; nhr-25* | - | 20.62 | 26.08 | 25.50 | 23.80 |
| *acs-20(tm3278)* | - | 30.55 | 11.55 | 11.55 | 15.28 |

|  | T-test P-value |
| --- | --- |
| WT vs *acs-3* | 1.18E-02 |
| WT vs *nhr-25* | 4.26E-01 |
| WT vs *acs-3 nhr-25* | 2.12E-02 |
| WT vs *acs-20* | 1.30E-02 |
|  |  |
| *acs-3 vs nhr-25* | 6.47E-02 |
| *acs-3 vs acs-3;nhr-25* | 7.69E-01 |
| *acs-3 vs acs-20* | 4.72E-02 |
|  |  |
| *nhr-25 vs acs-3;nhr-25* | 9.09E-02 |
| *nhr-25 vs acs-20* | 2.95E-01 |
|  |  |
| *acs-3;nhr-25 vs acs-20* | 2.05E-01 |
